# Mitral Valve Repair in a 15-Month-Old Child With Kingella kingae Endocarditis

**DOI:** 10.7759/cureus.63670

**Published:** 2024-07-02

**Authors:** Matthew D McGee, Sunni A Coyne, Renish N Contractor, Brian Winburn

**Affiliations:** 1 Pediatrics, Lake Erie College of Osteopathic Medicine, Bradenton, USA; 2 Pediatrics, Florida State University College of Medicine, Tallahassee, USA; 3 Pediatrics, AdventHealth Daytona Beach, Daytona Beach, USA

**Keywords:** septic emboli, pediatric, mitral regurgitation, kingella kingae, endocarditis

## Abstract

*Kingella kingae*, a *Haemophilus parainfluenzae, Aggregatibacter actinomycetemcomitans, Aggregatibacter aphrophilus, Cardiobacterium hominis, Eikenella corrodens, K. kingae* (HACEK) organism, is commonly found in the oropharynx. Although it rarely causes endocarditis, it can pose a significant risk to young children. We report a case of *K. kingae* endocarditis in a previously healthy 15-month-old male who initially presented with symptoms of an upper respiratory infection. Blood cultures taken at 60 hours revealed the presence of *K. kingae*. Subsequent echocardiogram and brain MRI demonstrated large vegetation on the mitral valve and septic emboli in the right occipital and left posterior parietal lobes. The patient was treated with intravenous ceftriaxone and underwent mitral valve repair with annuloplasty. This case illustrates the presentation of *K. kingae* endocarditis with initial respiratory symptoms and the subsequent identification of the infection through blood cultures and imaging. For pediatric patients presenting with upper respiratory symptoms, there may be clinical benefit to noninvasive ultrasound imaging to help rule out atypical pathologies like endocarditis.

## Introduction

The bacteria *Kingella kingae* (*K. kingae*) is a rare cause of endocarditis and pediatric heart disease. *K. kingae*, of the *Haemophilus parainfluenzae*, *Aggregatibacter actinomycetemcomitans*, *Aggregatibacter aphrophilus*, *Cardiobacterium hominis*, *Eikenella corrodens*, *K. kingae* (HACEK) group, is an encapsulated beta-hemolytic gram-negative coccobacilli of the *Neisseriaceae* family in the beta subclass of the *Proteobacteria* [1]. *K. kingae* can be a commensal organism in children’s oral cavities, however, can sometimes cause mild systemic disease and potential progression to endocarditis.

Here, we present a patient case of a 15-month-old African American male with an idiopathic development of *K. kingae* endocarditis of the mitral valve presenting as an upper respiratory infection (URI). This rare manifestation of infective endocarditis (IE) underscores the importance of early identification, particularly given its potential resemblance to various infectious illnesses in the pediatric demographic, in order to avert substantial disease progression.

## Case presentation

A 15-month-old African American male presented to the emergency department with his mother for flu-like symptoms of cough, congestion, and runny nose. He was sent home with a diagnosis of a URI. The patient returned five days later with a continuation of symptoms, fevers, decreased appetite, and fluid intake. Birth and medical history were unremarkable. The patient had no symptoms of vomiting, diarrhea, or rashes. Vaccinations were up to date. Family history was significant for an aunt with sickle cell disease, and the patient’s mother believed that the father was a carrier for the trait. The patient had a cousin who was recently admitted to the hospital for pneumonia with whom he was spending time with recently. The patient was given a dose of Ceftriaxone and a normal saline bolus in the emergency department. He was admitted to the hospital with a diagnosis of pneumonia due to respiratory distress, persistent fevers, and URI symptoms.

**Figure 1:**
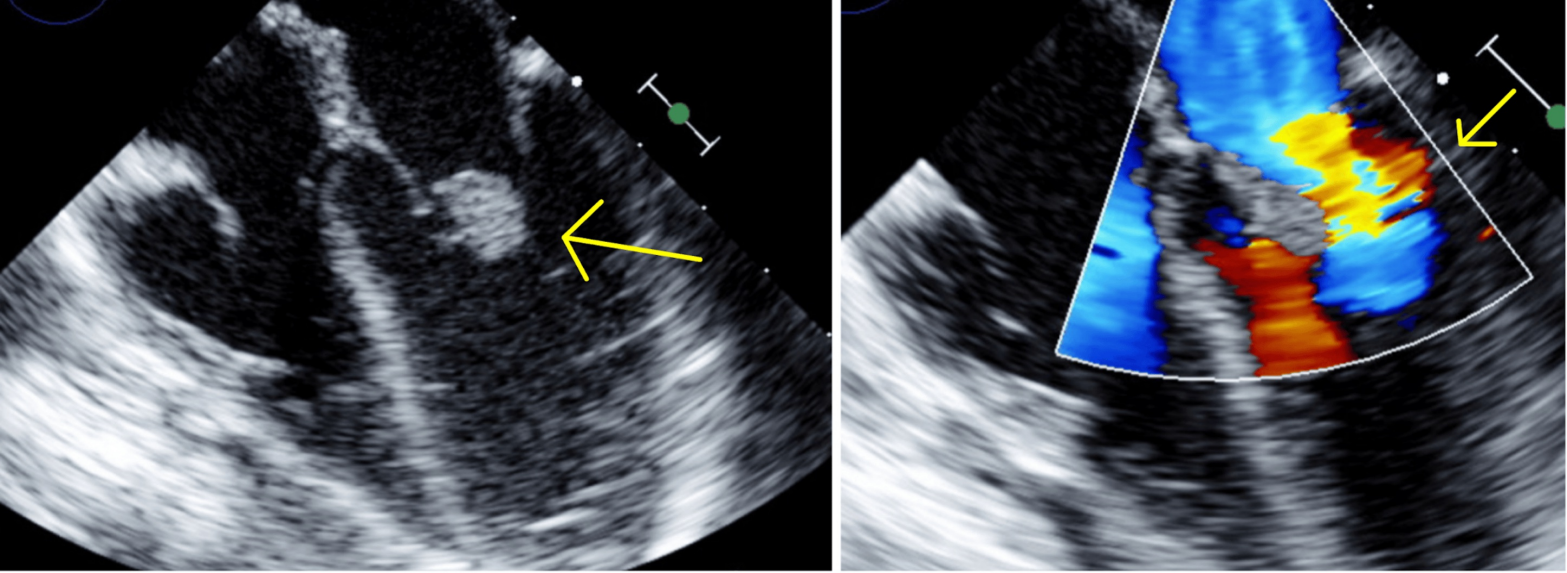
Transthoracic echocardiogram (TTE) images demonstrating a mitral valve mass (left) and regurgitation of blood flow (right).

On admission, the patient had a temperature of 103 degrees Fahrenheit, pulse of 158 beats per minute, respiratory rate of 58 breaths per minute, blood pressure of 101/78 mmHg, and SpO2 of 99% on room air. Physical exam was notable for decreased air movement in the left anterior lower lung field and shallow breathing. The rest of his physical exam was unremarkable. A CBC showed an elevated white count at 18.85 (4.00-13.70 × 10^3​​​​​^​​/µL), hemoglobin of 8.2 (10.2-13.0 g/dL), and normal platelet count at 223 (126-423 × 10^3^/µL). On the CMP, there was mild hyponatremia at 133 (136-145 mmol/L). Liver function tests were within normal limits, as well as PT/INR. Urinalysis was negative for any signs of infection. A respiratory viral panel was positive for Rhinovirus. The patient had an elevated CRP of 13 mg/L, and elevated procalcitonin at 1.91 (≤0.08 ng/mL). Blood cultures showed no growth in the first 24 hours. Continued observation at 60 hours showed growth of *K. kingae*, non-beta lactamase producing. Following the positive blood cultures, the patient was started on 75mg/kg IV Ceftriaxone. When his IV was lost, he was temporarily on oral amoxicillin for one day until IV access could be obtained, and IV Ceftriaxone could be restarted. He underwent a transthoracic echocardiogram (TTE) after a consultation with the pediatric infectious disease team. Results of the TTE showed a pedunculated mass on the anterior leaflet of the mitral valve measuring 1.7 cm x 0.6 cm with signs of mild regurgitation of the mitral valve (Figure 1). The patient had no immunologic findings or neurologic deficits. His oral intake of solids and liquids was decreased, otherwise he was voiding and sleeping well. He was hemodynamically stable and transferred to a higher level of care for surgical evaluation.

**Figure 2:**
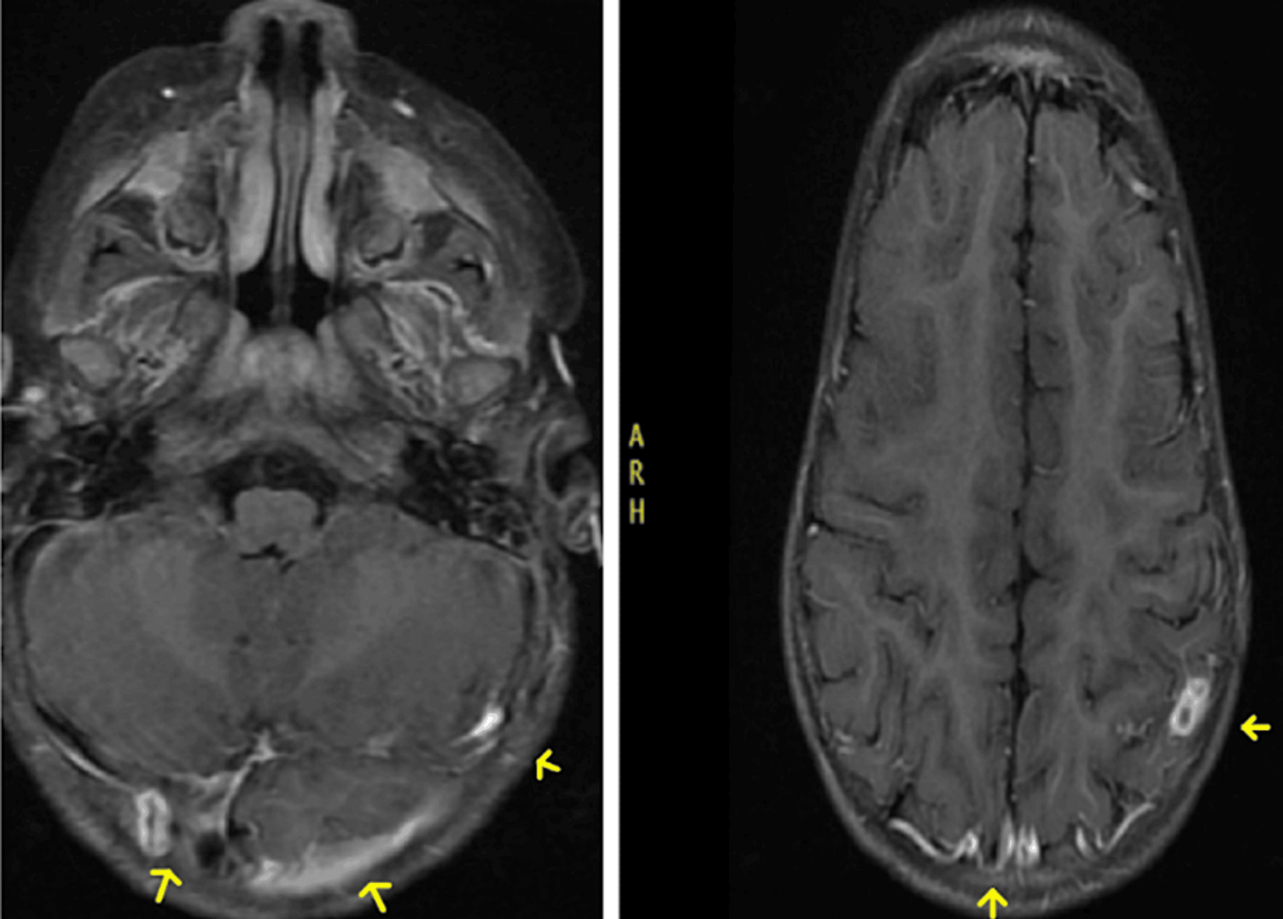
MRI showing right occipital and left posterior parietal peripherally enhancing lesions.

The patient was evaluated by cardiothoracic surgery who performed a mitral valve debridement on the patient. He underwent successful debridement of the vegetative mass, however on repeat echocardiogram during the procedure, there was severe mitral regurgitation and destruction to the mitral valve where the vegetation was attached to. As a result, the patient had a patch of Cardiocel bovine pericardium cut into the shape of the valve defect and sewn into the valve with annuloplasty, leaving only trivial mitral valve regurgitation, adequate biventricular function, and no residual ASD from the surgical process. The patient was transferred to the pediatric cardiac intensive care unit intubated and in stable condition. An MRI brain with and without contrast was ordered to look for possible septic emboli or infectious abscesses. Imaging showed right occipital and left posterior parietal peripherally enhancing lesions consistent with septic emboli given the patient’s history (Figure 2). The patient was continued on an increased ceftriaxone dose of 100 mg/kg/day IV every 24 hours. A peripherally inserted central catheter (PICC) line was placed for intravenous antibiotic administration after discharge from the hospital for 10 days, with PICC line removal six days afterward.

## Discussion

Pediatric IE is considered a rare phenomenon to encounter, especially so with HACEK organisms such as *K. kingae*. More commonly, *K. kingae* will present as pediatric septic arthritis, osteomyelitis, and/or bacteremia, after gaining access to the bloodstream through damage to the child’s oral mucosal. These cases tend to have a rather favorable prognosis than IE [2,3].

Endocarditis-related disease can cause complications such as valvular disease and possible septic embolization leading to downstream neurologic deficits [4]. Among children with IE, *S. aureus, S. viridans*, and other *Staphylococcus * and *Streptococcus* species comprise roughly 85% of infective organisms, with gram-negative bacilli and polymicrobial infections being the remaining 15%. In one study over an 11-year period from 2000 to 2010, using the Nationwide Inpatient Sample (NIS) database of children less than 19 years old, the incidence of IE had remained unchanged. However, cases of *Staphylococcus *species were declining while *Streptococcus* species were rising. Per the study, *Staphylococcus *endocarditis is associated with increased length of hospital stay and the highest mortality among bacteria [5]. *K. kingae* endocarditis affects the mitral valve in over 90% of cases (particularly in the posterior leaflet), followed by the aortic valve, and is rare in the tricuspid or pulmonic valve [6].

Although uncommon, with incidence rates of 0.3 to 3.3 per 100,000 children each year, IE should not be overlooked as a diagnosis in children due to the significant risk of morbidity and mortality. Further, the majority of patients who develop IE in the pediatric setting have underlying comorbidities such as congenital heart disease or complications of rheumatic heart disease. Around 10% of children with IE have no underlying conditions or comorbidities [7]. 

For diagnosis of IE, historically the Duke criteria have been used. In 2023, the International Society for Cardiovascular Infectious Disease (ISCVID) updated their criteria from their modified criteria going back to the year 2000 (Table 1). The new 2023 criteria showed higher sensitivity (84%) compared to previous versions (70%). However, the specificity of the new clinical criteria was lower (60%) compared to prior criteria (74%) [8]. The criteria were updated to address changes in epidemiology and the use of modern imaging and diagnostic testing. In the pediatric setting, the Duke criteria are still used; however, diagnosis of IE is complex due to nonspecific manifestations of illness which often get overlooked for common conditions, and need for elevated clinical suspicion is required [9].

**Table 1:** 2023 ISCVID Duke major and minor criteria ISCVID: International Society for Cardiovascular Infectious Disease

Criteria
Major criteria
1. Positive blood cultures or microbiologic testing (PCR/IgG titers/immunofluorescence assays)
Two or more blood cultures drawn 12 hours apart
Three or majority of ≥ 4 separate blood cultures, ≥ 1 hour from first to last
2. Imaging evidence of endocardial involvement
Positive echocardiography and/or CT/PET imaging
3. Added Surgical criteria of evidence of endocardial/valve involvement during cardiac surgery
Minor criteria
1. Predisposing heart condition/ IV drug use
2. Fever, temperature >38 degrees celcius
3. Vascular phenomena*
4. Previous history of IE
5. Prosthetic valve or history of valve repair
6. Congenital heart disease
* Evidence of emboli, infarct, cerebral/splenic abscess, ICH, conjunctival hemorrhages, Janeway lesions, purpura
Confirmed infective endocarditis
2 major criteria
1 major + 3 minor criteria
5 minor criteria
Possible infective endocarditis
1 major + 1 minor criteria
3 minor criteria

The initial presentation of our patient with flu-like symptoms and an initial diagnosis of an URI exemplifies the challenges in recognizing pediatric IE at its onset, especially in previously healthy children. This delay in diagnosis is not uncommon, and the potential for serious complications, as seen in this case, necessitates a high index of suspicion and a more comprehensive diagnostic approach. Further, previously healthy children who are found to have IE tend to have a worse prognosis and an increased risk of complications compared to those with an identifiable predisposing condition [9]. One possible reason for this is the reliance on heuristics and anchoring bias in clinical decision-making that can lead to a delay in diagnosis or misdiagnosis for atypical presentations like our case. Furthermore, and likely more importantly, *K. kingae* is known as a “culture-negative” bacteria, making it difficult to identify on cultures due to minimal growth and long incubation times [10]. In our patient, there was no growth of blood cultures at 24 hours; however, they were followed until 60 hours, which resulted in the growth of *K. kingae* and prompted the remainder of treatment, including a stat echocardiogram and subsequent mitral valve repair. Mitral valve replacement in the pediatric population, especially younger than two years of age, is associated with decreased long-term survival rates with five and 10-year survival rates of 33% to 95%. The mitral valve has been shown to have the highest rate of mortality and poor long-term prognosis among pediatric valve replacements and since the valves are fixed in size, it is expected that repeat valve replacements will be necessary [6,11].

The case further portrays the insidious onset and challenges of diagnosing pediatric IE. Infection with *K. kingae* poses an even greater challenge due to its slow growth on blood cultures, which further delays the diagnosis and timing of treatment. Looking back on the case, since blood cultures are not routinely obtained for mild upper respiratory symptoms, it remains unknown whether or not our patient’s initial presentation was due to the Rhinovirus URI or the* K. kingae* infection, or a combination of both. Up until 60 hours after blood cultures were obtained and initially negative, our only positive organism to treat for was Rhinovirus from a respiratory panel. Our patient was showing signs of initial improvement prior to the culture results other than the refusal of PO intake of solids and liquids, giving time for growth on the blood cultures that came back positive almost three days later for *K. kingae* that we were able to subsequently intervene against.

Timing is arguably most important in these cases and yet, it is routine to await the results of the blood culture to provide definitive treatment. Implementing noninvasive ultrasound imaging such as an echocardiogram as part of the clinical workup of respiratory infections may show clinical benefit in ruling out rare conditions without causing harm to the patient.

## Conclusions

In this paper, we report a case of a healthy toddler with no previous cardiac history who developed *K. kingae* endocarditis of the mitral valve and subsequent septic emboli to the occipital and parietal lobes. *K. kingae* is a common colonizer of the oropharynx and can rarely cause invasive infections. Such infections typically occur in children between the ages of six months and four years and involve the joints, bones, and endocardium. The patient's presentation of flu-like symptoms and the delayed growth of *K. kingae* on blood cultures highlight the insidious onset of the infection and how this interferes with timely diagnosis and early treatment to prevent adverse long-term effects. Incorporating ultrasound imaging, such as echocardiograms, into the clinical evaluation of respiratory infections may help identify rare conditions early without posing any risk to the patient.
